# Dupilumab-Associated Severe Headaches in Chronic Rhinosinusitis With Nasal Polyposis (CRSwNP): A Report of Two Cases With Resolution After Cessation

**DOI:** 10.7759/cureus.109262

**Published:** 2026-05-20

**Authors:** Michael Wang, Sonya Marcus

**Affiliations:** 1 Otolaryngology-Head and Neck Surgery, Stony Brook University, Stony Brook, USA

**Keywords:** adverse drug reaction, biologic therapy, chronic rhinosinusitis with nasal polyposis, dupilumab, headache, monoclonal antibody

## Abstract

Dupilumab is a monoclonal antibody that targets interleukin (IL)-4 and IL-13, approved for the treatment of chronic rhinosinusitis with nasal polyposis (CRSwNP). While common adverse effects such as conjunctivitis and injection-site reactions are well characterized, headaches reported in clinical trials are typically described as mild, non-specific, and rarely treatment-limiting. We describe two patients with CRSwNP who developed severe, recurrent headaches shortly after initiating dupilumab therapy. In one case, symptoms improved after discontinuation and recurred upon rechallenge; in the other, the headaches resolved after discontinuation. These observations raise the possibility that dupilumab-associated headaches may occasionally be more severe than previously appreciated. Clinicians should be aware of this potential association when evaluating new-onset headaches in patients receiving dupilumab.

## Introduction

Chronic rhinosinusitis with nasal polyposis (CRSwNP) is a type 2 inflammatory disorder characterized by nasal obstruction, anosmia, and chronic sinonasal mucosal inflammation [1]. For patients with refractory disease, treatment options have expanded with the introduction of dupilumab, a fully human monoclonal antibody targeting the interleukin-4 receptor alpha (IL-4Rα) subunit, thereby inhibiting both IL-4 and IL-13 signaling [2]. Dupilumab has demonstrated substantial efficacy in improving disease-specific quality of life in patients with CRSwNP, and its overall tolerability profile is well established.

Among the adverse effects reported in the pivotal phase III LIBERTY NP SINUS-24 and SINUS-52 trials, headaches occurred at rates comparable to or slightly lower than placebo, approximately 7% versus 9%, respectively, and were not further characterized with respect to severity, duration, or clinical impact [3]. This aggregate reporting reflects the nature of large, randomized trials, in which adverse events are systematically captured but may not fully convey the range of presentations encountered in clinical practice.

Here, we present two patients with CRSwNP who developed severe, treatment-limiting headaches shortly after initiating dupilumab. In one case, symptoms improved after discontinuation and recurred upon rechallenge; in the other, headaches resolved completely after stopping therapy. These cases raise the possibility that dupilumab-associated headaches may occasionally exceed the mild, transient presentations captured in trial data and highlight the importance of considering this potential association in clinical practice.

## Case presentation

Case 1

A 68-year-old man with CRSwNP, severe persistent asthma, allergic rhinitis, and a remote history of endoscopic sinus surgery was initiated on dupilumab for refractory sinonasal symptoms. His medical history was also notable for rheumatoid arthritis and cervical spinal stenosis. Concomitant medications included albuterol, azelastine nasal spray, tamsulosin, fluticasone propionate nasal spray, icosapent ethyl, montelukast, and budesonide-formoterol.

Three weeks after treatment initiation, he developed new recurrent headaches described as a burning, radiating sensation across the forehead. Over the ensuing six months, the headaches progressed from mild discomfort to severe pain. He denied associated visual disturbances, photophobia, nausea, or vomiting. Over-the-counter ibuprofen provided no meaningful relief.

Nasal endoscopy and computed tomography (CT) demonstrated bilateral grade II polyps without purulence or other findings suggestive of acute sinusitis (Figure 1). Given his history of rheumatoid arthritis and concurrent xerophthalmia and xerostomia, he underwent rheumatologic evaluation for possible secondary Sjögren syndrome, but the workup was unremarkable. Neurologic evaluation raised the possibility of new-onset migraine, but the patient declined pharmacologic treatment. Magnetic resonance imaging (MRI) of the brain was unremarkable. His known cervical spinal stenosis and chronic neck pain were also considered as a potential contributing etiology; however, they are considered less likely in the context of the temporal relationship to dupilumab initiation.

**Figure 1:**
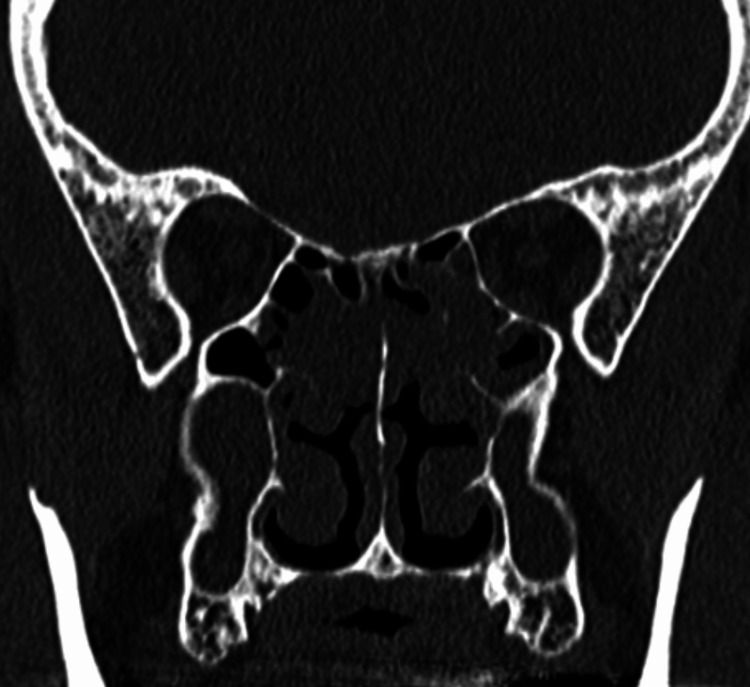
Coronal CT of the paranasal sinuses demonstrating bilateral ethmoid and maxillary sinus mucosal thickening. CT: computed tomography

Given the temporal association between dupilumab initiation and headache onset, dupilumab was discontinued, with subsequent improvement in symptoms. Because of the clinical benefit of dupilumab in controlling his type 2 inflammatory disease, therapy was later resumed, after which headaches of the same character recurred.

Case 2

A 41-year-old man with CRSwNP was started on dupilumab for refractory disease. His surgical history was notable for prior bifrontal craniotomy and frontal sinus obliteration performed for repair of a traumatic cerebrospinal fluid (CSF) leak. He had no prior history of a headache disorder, migraine, or intracranial hypertension and was headache-free at baseline before dupilumab initiation. Concomitant medications included albuterol, atorvastatin, lamotrigine, and melatonin.

Within seven days of the first dupilumab dose, he developed new-onset severe, paroxysmal headaches occurring two to three times per week. He denied nausea, vomiting, photophobia, or focal neurologic symptoms. Symptoms were only partially responsive to nonsteroidal anti-inflammatory drugs (NSAIDs). CT of the sinuses showed pansinusitis (Figure 2), and nasal endoscopy demonstrated bilateral grade III polyps without purulence or findings of acute sinusitis. Although his prior craniotomy and frontal sinus obliteration raised the consideration of a post-surgical headache etiology, the patient had been consistently headache-free in the interval since his surgery and remained so until dupilumab initiation, making this explanation less compelling.

**Figure 2:**
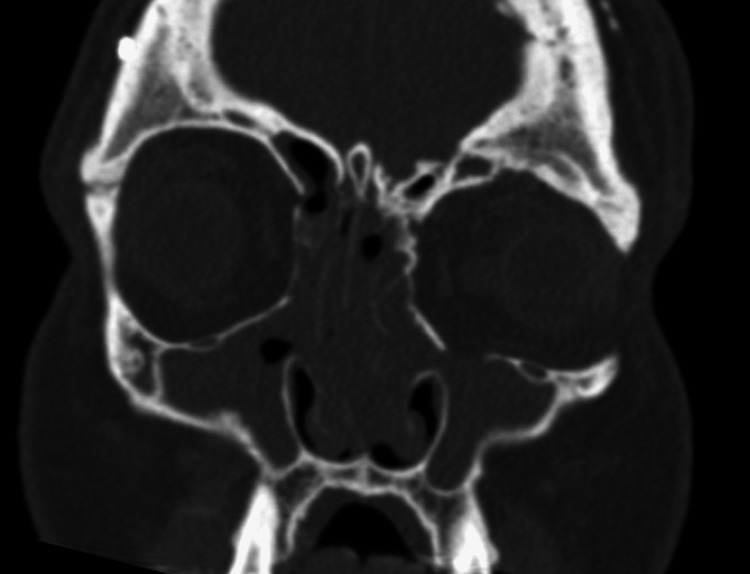
Case 2: coronal CT of the sinuses demonstrating diffuse sinonasal disease and post-surgical changes of the left frontal sinus following obliteration for traumatic CSF leak repair. CT: computed tomography; CSF: cerebrospinal fluid

Given the close temporal relationship between dupilumab initiation and headache onset, the medication was discontinued. Neurologic evaluation was not completed, as the patient attributed the headaches to dupilumab and elected to stop therapy before consultation could be arranged. The headaches resolved completely within four weeks of the last dose, with gradual improvement during that interval. Rechallenge was not performed because of patient preference, and alternative management strategies for CRSwNP were subsequently pursued.

Table 1 provides the clinical characteristics and headache course in two patients with CRSwNP following dupilumab initiation.

**Table 1:** Clinical characteristics and headache course in two patients with CRSwNP following dupilumab initiation. CRSwNP: chronic rhinosinusitis with nasal polyposis; CT: computed tomography; CSF: cerebrospinal fluid; NSAIDs: nonsteroidal anti-inflammatory drugs; MRI: magnetic resonance imaging; OTC: over the counter

	Case 1	Case 2
Age/sex	68 years/male	41 years/male
Indication	CRSwNP, severe persistent asthma	CRSwNP
Relevant comorbidities	Rheumatoid arthritis, cervical spinal stenosis	Prior bifrontal craniotomy and frontal sinus obliteration for traumatic CSF leak
Concomitant medications	Albuterol, azelastine nasal spray, tamsulosin, fluticasone propionate nasal spray, icosapent ethyl, montelukast, budesonide-formoterol	Albuterol, atorvastatin, lamotrigine, melatonin
Time to headache onset	~3 weeks after the first dose	7 days after the first dose
Headache character	Burning, radiating; frontal distribution; progressive over 6 months from mild to severe	Severe, paroxysmal; 2-3 episodes per week
Associated symptoms	None	None
Analgesic/migraine therapy	OTC ibuprofen (no meaningful relief); pharmacologic migraine therapy declined	NSAIDs (partial response only)
Diagnostic workup	MRI of the brain (unremarkable); CT of the sinuses + nasal endoscopy (grade II polyps, no purulence); rheumatology evaluation for Sjögren syndrome (unremarkable)	CT of the sinuses (pansinusitis); nasal endoscopy (grade III polyps, no purulence); neurologic evaluation not completed
Alternative etiologies considered	Cervicogenic (less likely; symptoms arose only after dupilumab initiation); new-onset migraine (raised by neurology); secondary Sjögren syndrome (excluded)	Post-craniotomy headache (less likely; patient headache-free from surgery until dupilumab initiation)
Response to discontinuation	Improved	Complete resolution within 4 weeks
Rechallenge	Yes (headaches recurred)	Not performed (patient preference)

## Discussion

Dupilumab has emerged as a key therapeutic option for patients with refractory CRSwNP. In the pivotal phase III LIBERTY NP SINUS-24 and SINUS-52 trials, headaches were reported slightly more frequently in the placebo group than in the dupilumab cohort (approximately 9% vs. 7%), and the published trial reports did not further characterize the nature, severity, or clinical relevance of these events [3]. To our knowledge, this is the first case series describing severe, treatment-limiting headaches in patients with CRSwNP temporally associated with dupilumab initiation.

In both cases, symptoms were temporally linked to the start of therapy and were refractory to conservative management. In case 1, headaches improved after discontinuation and recurred upon rechallenge, whereas in case 2, symptoms resolved within one month of discontinuation. Together, these temporal patterns, and particularly the rechallenge-recurrence sequence in case 1, support a possible drug-related association. Importantly, both individuals had comorbid conditions that could also have contributed to headache etiology: cervical spinal stenosis in case 1 and a prior bifrontal craniotomy for traumatic CSF leak in case 2. In case 1, however, the cervicogenic etiology was considered less likely given that symptoms emerged only after dupilumab initiation. In case 2, the patient was headache-free at baseline prior to starting dupilumab, making a post-craniotomy etiology similarly less compelling. Accordingly, causality cannot be definitively established, but the clinical course in both cases raises concern for a dupilumab-associated headache syndrome.

The mechanism underlying such severe headache remains unclear. IL-4 and IL-13 are pleiotropic cytokines whose signaling extends beyond the peripheral immune system. Receptors for both cytokines have been identified on neurons and glial cells in preclinical models, suggesting a role in central neuroimmune regulation [4]. IL-4 has been shown to modulate microglial activation and neuroinflammatory cascades in rodent models [5], and IL-13 signaling has been implicated in the regulation of nociceptive pathways [6]. Inhibition of these pathways by dupilumab could therefore plausibly alter central sensitization or pain modulation thresholds, particularly in individuals with pre-existing neurologic vulnerabilities. Alternatively, dupilumab may unmask an underlying migraine susceptibility in susceptible patients by shifting the cytokine milieu in a way that lowers the threshold for trigeminovascular activation [7].

The present report has several limitations inherent to its retrospective, electronic medical record (EMR)-based design. Although concomitant medications were identified for both patients, a complete medication reconciliation could not be confirmed from available records, and the possibility of drug interactions contributing to headaches cannot be fully excluded. Notably, lamotrigine in case 2 is a centrally active agent that may influence neurologic thresholds, and its role cannot be definitively ruled out. In case 2, neurologic evaluation was not completed prior to discontinuation, leaving the headache phenotype incompletely characterized. Additionally, with only two cases, the generalizability of these observations is necessarily limited, and the incidence of severe dupilumab-associated headache in the broader CRSwNP population remains unknown. These cases nonetheless highlight the importance of recognizing severe headache as a potential, albeit uncommon, adverse reaction to dupilumab. Clinicians should maintain awareness of this possible association when new or worsening headaches occur after therapy initiation, particularly in patients with complex comorbidities or neurologic histories.

## Conclusions

While dupilumab is generally well tolerated and highly effective in managing CRSwNP, these cases raise the possibility that severe, treatment-limiting headaches may occur in rare instances. Recognition of this potential association is clinically important, particularly when symptoms arise shortly after treatment initiation and improve after discontinuation or recur upon rechallenge.
